# Indole 3-acetate and response to therapy in borderline resectable or locally advanced pancreatic cancer

**DOI:** 10.3389/fonc.2024.1488749

**Published:** 2024-12-20

**Authors:** Peder R. Braadland, Ingvild Farnes, Elin H. Kure, Sheraz Yaqub, Adrian McCann, Per M. Ueland, Knut Jørgen Labori, Johannes R. Hov

**Affiliations:** ^1^ Research Institute of Internal Medicine and Norwegian PSC Research Center, Division of Surgery and Specialized Medicine, Oslo University Hospital, Oslo, Norway; ^2^ Institute of Clinical Medicine, University of Oslo, Oslo, Norway; ^3^ Department of Hepato-Pancreato-Biliary Surgery, Oslo University Hospital, Oslo, Norway; ^4^ Department of Cancer Genetics, Institute for Cancer Research, Oslo University Hospital, Oslo, Norway; ^5^ Department of Natural Sciences and Environmental Health, University of South-Eastern Norway, Bø i Telemark, Norway; ^6^ BEVITAL AS, Bergen, Norway; ^7^ Department of Clinical Science, University of Bergen, Bergen, Norway; ^8^ Section of Gastroenterology, Department of Transplantation Medicine, Oslo University Hospital, Oslo, Norway

**Keywords:** pancreatic adenocarcinoma, chemotherapy - oncology, biomarkers, microbiome, indoles, 3-IAA, indole 3-acetic acid

## Abstract

**Background/Aims:**

It was recently reported that a higher concentration of the bacterially produced metabolite indole 3-acetate (3-IAA) in blood was linked to a better response to chemotherapy in patients with metastatic pancreatic ductal adenocarcinoma (PDAC). Here, we aimed to extend these observations to patients diagnosed with non-metastatic PDAC.

**Method:**

We measured circulating 3-IAA in samples from a prospective population-based cohort of 124 patients with borderline resectable or locally advanced PDAC, collected before initiating neoadjuvant chemotherapy. The majority (61%) of the patients were treated with FOLFIRINOX. We used univariable and multivariable Cox proportional hazards regression to estimate the association between pre-treatment 3-IAA and overall survival.

**Results:**

The median serum 3-IAA concentration before chemotherapy was 290 (interquartile range 203–417) ng/mL. The unadjusted hazard ratio (HR) for pre-treatment log_2_(3-IAA) was 0.93, 95% confidence interval (CI) [0.74–1.16], p=0.52. When adjusting for age, ECOG, CA19-9 and tumor classification, the HR for log_2_(3-IAA) was 0.87, 95% CI [0.68–1.12], p=0.28.

**Conclusion:**

Our findings suggest that the potentiating effect of 3-IAA observed in metastatic PDAC undergoing chemotherapy may not translate to borderline resectable or locally advanced PDAC. We recommend additional clinical validation of 3-IAA’s predictive value in different categories of PDAC before implementation attempts in human studies are initiated.

## Introduction

Pancreatic ductal adenocarcinoma (PDAC) is one the most lethal cancers, with an incidence rate of about 15 per 100 000 per year in Norway (2023) (1). About 85% of patients with PDAC are not eligible for upfront surgery with curative intent. Many of these receive neoadjuvant or palliative therapy, but unfortunately, the response rates are low (2). Biomarkers of chemotherapy response are crucial for selecting patients who are likely to benefit from treatment in a personalized way (3).

In PDAC, carbohydrate antigen 19-9 (CA19-9) is the most commonly used biomarker for therapy response, but has limited predictive power (4). Considering new methods in oncology in general, tumor characteristics like expression of specific receptors or the presence of gene rearrangements have so far been the typical candidates as biomarkers (3). The gut microbiota composition has been increasingly associated with multiple health characteristics, including the response to some cancer therapies (5, 6). Recently, Tintelnot et al. identified circulating levels of the gut microbial metabolite indole 3-acetate (3-IAA) as positively associated with response to chemotherapy in metastatic pancreatic ductal adenocarcinoma (PDAC) (7). In an elegant experimental and molecular characterization study, the mechanism of increased chemotherapeutic effect was found to depend on oxidation of 3-IAA by myeloperoxidase from neutrophils, which subsequently increased reactive oxygen species in tumor cells and hence tumor cell death. The study introduced a novel concept, whereby a metabolite produced by gut microbes is absorbed systemically and modifies chemotherapy response in the host (8). In the study, chemotherapy response in mice could even be improved by supplementing the diet with the 3-IAA precursor tryptophan, leading to increased 3-IAA levels, suggesting that a similar adjuvant approach may demonstrate clinical efficacy in humans.

Chemotherapy in metastatic PDAC is given with palliative intent, but it is the primary treatment for patients with borderline resectable or locally advanced PDAC in order to improve resection rates with curative intent (2). We therefore measured 3-IAA in a cohort of patients with borderline resectable or locally advanced PDAC before starting chemotherapy (9), aiming to investigate if elevated serum 3-IAA associates with therapeutic response in a PDAC population without distant metastases.

## Patients and methods

### Study design and participants

The participants included in this study were from the NORPACT-2 study (9), which was a prospective, population-based cohort of patients with borderline resectable or locally advanced PDAC enrolled at the Oslo University Hospital between 2018 and 2020. We included 124 patients who had donated blood before initiation of any chemotherapy (flowchart shown in **Supplementary Figure S1**). A subset of 35 (28%) patients who were available and willing to donate additional samples had a second blood draw 2–4 months after initiation of chemotherapy. The study protocol was approved by the Regional Ethical Committee of Medical and Health Research Ethics Nord 2017/1382. To assess population concentrations of 3-IAA, n=100 healthy control serum samples (median age 40, female 41%) studied in the context of other studies were also included (10), approved by the Regional Ethical Committee of Medical and Health Research Ethics South-Eastern Norway 2015/2140.

### Outcome, definitions and diagnostic criteria

Borderline resectable or locally advanced PDAC was diagnosed according to the National Comprehensive Cancer Network (NCCN) criteria, version 2, 2017 (2). Distant metastases were ruled out using computed tomography (CT) scans of the abdomen and chest. Fine-needle aspiration cytology or biopsy by endoscopic ultrasound was required to confirm PDAC. The chemotherapy regimen was decided by the treating medical oncologist at the local hospital.

The primary outcome was defined as overall survival, and time to the outcome was defined as the time from blood draw, which was done in conjunction with initiation of primary chemotherapy. A secondary outcome was included where participants were censored at their time of surgical resection (after start of primary chemotherapy) where applicable.

### Targeted liquid chromatography-tandem mass spectrometry

Baseline and follow-up serum was sampled and stored according to a standardized procedure at Oslo University Hospital. Serum was left to coagulate for at least 30 minutes, followed by centrifugation at 2450 x g for 15 minutes and frozen and stored at -80°C. Targeted metabolite analysis of 3-IAA was performed using a liquid chromatography-tandem mass spectrometry (LC-MS/MS) platform, which is also used for measuring B-vitamins and human and bacterial tryptophan metabolites at BEVITAL (www.bevital.no) as described in Midttun et al. (11).

Briefly, 3-IAA was added to the established assay using indole-2,4,5,6,7-d_5_-3-acetic acid (3-IAAd_5_), obtained from C/D/N Isotopes Inc (Quebec, Canada), as internal standard. The retention times were 4.93 min (3-IAA) and 4.91 min (3-IAAd_5_). The analytes were detected in positive-ion multiple reaction monitoring (MRM) mode, using the ion-pairs of 176.2/130.2 m/z and 181.1/134.1 m/z for 3-IAA and 3-IAAd_5_, respectively.

### Statistics

Surviving proportions were visualized by plotting Kaplan-Meier survival curves of quartiles of 3-IAA. Cox proportional hazards models were used to estimate 3-IAA’s (log_2_-transformed) association with overall survival. We included ECOG performance status (modeled as a continuous variable), CA19-9 (log_2_-transformed), tumor classification (locally advanced or borderline resectable) and age in a multivariable Cox model.

To test for differences in distributions of continuous variables we used the Wilcoxon rank-sum test for independent observations and the Wilcoxon signed-rank test for paired observations. Distributions are given as median (interquartile range, IQR).

For any variable used in the analyses, data were missing in less than 5% of the participants and missing data were therefore not imputed. All data processing, visualization and statistical analyses were performed in R.

## Results

In total, 124 patients (median 68 years, 47% male) with borderline resectable (n=68) or locally advanced PDAC (n=56) were included (**Table 1**). Seventy-six (61%) patients were treated with fluorouracil, leucovorin, irinotecan, and oxaliplatin (FOLFIRINOX), 26 (21%) with gemcitabine plus nab-paclitaxel, and 22 (18%) with gemcitabine monotherapy or other chemotherapy regimens.

**Table 1:** Baseline characteristics of the study population.

Characteristics	N = 124
Age	68 (60, 73)
Sex, male	58 (47%)
Body mass index, median (IQR)	23.9 (21.0, 27.0)
Performance status (ECOG)	
0	80 (65%)
1	40 (32%)
>1	4 (3.2%)
Charlson comorbidity index (- for tumor/age)	
0	62 (50%)
1	43 (35%)
>1	19 (15%)
Anatomic tumor classification	
Borderline resectable	68 (55%)
Locally advanced	56 (45%)
CA19-9, kU/L	289 (52, 825)
0-500 kU/L	70 (59%)
500-1000 kU/L	19 (16%)
≥1000 kU/L	20 (17%)
Non-producer	7 (5.9%)
Hyperbilirubinemia	2 (1.7%)
Missing	6 (4.8%)
Biliary stent	67 (54%)
Tumor location	
Head	101 (81%)
Body/Tail	23 (19%)
Tumor size (CT), mm	35 (29, 43)
Primary chemotherapy	
Fluorouracil, leucovorin, irinotecan, and oxaliplatin	76 (61%)
Gemcitabine plus nab-paclitaxel	26 (21%)
Gemcitabine monotherapy	18 (15%)
Other	4 (3%)

Continuous variables are shown as median (IQR). CA19-9, Carbohydrate antigen 19-9; ECOG, Eastern Cooperative Oncology Group.

The median 3-IAA concentration before chemotherapy was 290 (IQR 203–417) ng/mL. This was within the range of concentrations observed in n=100 healthy individuals analyzed in the context of another study (median 377 (IQR 303–464) ng/mL). Serum 3-IAA was positively correlated with age (Spearman’s ρ=0.33, p<0.001; **Supplementary Figure S2A**) but not with tumor diameter or serum CA19-9 (**Supplementary Figures S2B, C**). Pre-treatment 3-IAA was similar in the different chemotherapy groups (**Supplementary Figure S2D**).

**Figure 1:**
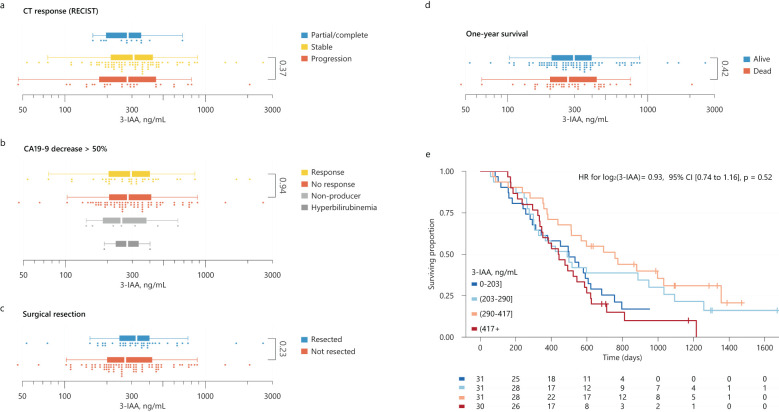
**(A–D)** Distributions of 3-IAA by CT-response according to RECIST criteria **(A)**, CA19-9 decrease > 50% **(B)**, whether surgical resection after neoadjuvant chemotherapy was performed or not **(C)**, and by one year survival status **(D)**. Statistical significance was tested using Mann-Whitney U tests. **(E)** Kaplan-Meier survival curves for overall survival for participants categorized by their pre-treatment 3-IAA concentration (quartiles) with number at risk below the plot. The result from a univariable Cox model for log(3-IAA) is shown as inset in the plot. 3-IAA, indole 3-acetate; CI, confidence interval; HR, hazard ratio.

We found no statistically significant differences in pre-treatment 3-IAA concentrations according to response as defined by CT scan or CA19-9 decline (**Figures 1A, B**). The 3-IAA concentration was numerically higher in patients who later had surgical resection and in those alive after one year, but the difference was not statistically significant (**Figures 1C, D**). In participants with a second sample taken, 3-IAA concentrations were on average increased after chemotherapy (median 265 nmol/L before and 328 nmol/L after), but the increase was not statistically significant (p=0.24, **Supplementary Figure S2E**).

The median survival time was 499 days (95% confidence interval (CI) 437–597). Twenty-six (21%) patients were event-free at their last follow-up date. We stratified participants into quartiles by pre-treatment 3-IAA concentrations but found no significant differences in survival between the groups (**Figure 1E**), a finding supported by a Cox model where 3-IAA (log_2_) had a HR=0.93, 95% CI [0.74–1.16], p=0.52. The model estimates were similar in the subgroups of borderline resectable or locally advanced cancer. Since 41 (33%) of the participants eventually had surgical resection of their primary tumor, which could influence survival, we evaluated the prognostic value of 3-IAA when censoring these cases at their time of surgery. Also here, we found no evidence of an association between 3-IAA and overall survival (**Supplementary Figure S3**). Finally, using the full cohort, we adjusted 3-IAA for age, ECOG, CA19-9 and tumor classification, where 3-IAA (log_2_) had an adjusted HR=0.87, 95% CI [0.68–1.12], p=0.28.

## Discussion

In the present study, serum 3-IAA concentration was not associated with response to chemotherapy or overall survival in a population-based cohort of borderline resectable or locally advanced PDAC starting chemotherapy. The results suggest that high 3-IAA levels do not predict chemotherapy response in borderline resectable or locally advanced PDAC.

The only data available for comparison are from the seminal paper by Tintelnot and co-workers (7) where an association between 3-IAA and chemotherapy response in PDAC was found. There are several differences between these studies that could potentially explain these conflicting observations. The association between chemotherapy response and 3-IAA in the Tintelnot et al. study was investigated in a total of 47 patients divided on two cohorts of metastatic PDAC (9). In contrast, while we studied more than the double number of patients (n=124), our population had no signs of distant metastasis. Patients with non-metastatic cancers often have a smaller total tumor load, better functional status (12), and differences in tumor biology compared with metastatic cancers (3, 13), all of which may influence the effect of chemotherapy and its modifiers.

Another major difference is the analytical methods. The median 3-IAA concentration in the present study was about 10-fold higher than what was found in Tintelnot et al. In a healthy control population analyzed in the context of other ongoing studies, the median 3-IAA was within the same order of magnitude. We therefore speculate that the discrepancy in 3-IAA concentrations arose mainly from assay differences, and it is difficult to exclude that this may in part contribute to the diverging results. Notably, the present data were generated with high quality LC-MS/MS-based methodology with a radioactively labelled internal standard. Nonetheless, if inter-individual variation in 3-IAA measurements was consistent with the two methods, we would expect to find an association with survival in our cohort if it truly existed. In our univariable model, 3-IAA had a wide confidence interval (0.74–1.16). While this makes it challenging to confidently assert the presence or absence of an effect, any potential clinical significance appears most likely to be small.

A third point is that apparent predictive performances in discovery cohorts are often optimistic and models commonly perform worse in new populations for a variety of reasons (14). This is illustrated in the study from Tintelnot et al. where the effect size observed in the second human PDAC cohort was lower than in the first cohort (explained variance in the correlation between 3-IAA and survival time was R^2^ = 0.51 in a cohort from Hamburg and R^2^ = 0.24 in a cohort from Munich).

One of the imitations of our study is that 54% of the patients received a biliary stent due to jaundice, and it remains unclear whether hyperbilirubinemia influences the gut metabolism of tryptophan, the precursor of 3-IAA (15, 16). Furthermore, only 28% of the cohort had a second blood sample withdrawn for serum 3-IAA measurement and only 61% were treated with FOLFIRINOX.

In conclusion, our results do not align with the optimism generated by Tintelnot et al.’s study. Additional validation of 3-IAA as a biomarker of chemotherapy response in PDAC is necessary before initiating human clinical trials aiming to modify 3-IAA levels in this setting. Furthermore, subsequent research in this field should probably focus on metastatic PDAC.

## Data Availability

Data are not deposited in a public repository due to data privacy regulations in Norway and lack of participant consent. However, data are available on request, if the aim of the analysis is covered by the consent signed by the participants, following an amendment to the ethics approval and a data transfer agreement. Requests to access the datasets should be directed to JH, j.e.r.hov@medisin.uio.no.

## Supplementary material

Supplementary Figure 1 Flow chart showing standard-of-care for this patient group and the timing of blood samples in the present study.

Supplementary Figure 2
**(A–C)** Plots of baseline 3-IAA concentrations versus age, largest tumor diameter (by CT-scan) and CA19-9, all measured at baseline (before primary chemotherapy). The bivariate associations were tested using Pearson’s (r) and Spearman’s (ρ) correlation. The red lines show the linear regression fits with shaded 95% confidence intervals. **(D)** Distribution of pre-treatment 3-IAA by chemotherapy regimen (FOLFIRINOX versus all other). Statistical significance was tested using a Mann-Whitney U test. **(E)**. Distributions of 3-IAA before and after primary chemotherapy, with lines indicating changes from before to after. Statistical significance was tested using a paired Wilcoxon signed-rank test. 3-IAA, 3-indoleacetic acid; FFX, FOLFIRINOX.

Supplementary Figure 3 Kaplan-Meier survival curves for overall survival, censoring participants who underwent surgical resection at their times of surgery. Patients were categorized by their baseline 3-IAA concentration (quartiles). The number of patients at risk at the indicated time points is shown below the plot. The results from a univariable Cox model for log2(3-IAA) is shown as an inset. 3-IAA, indole 3-acetate; CI, confidence interval; HR, hazard ratio.

## References

[1] Larsen IK Johannesen TB Seglem AH Hestad JJ Jakobsen E Mangrud OM. Cancer in Norway 2023. In: Larsen IK, editor. Cancer Registry of Norway (Kreftregisteret) (2024). M. BR.T. E., L. S.

[2] Tempero MA Malafa MP Al-Hawary M Behrman SW Benson AB Cardin DB. Version 2.2021, NCCN clinical practice guidelines in oncology. J Natl Compr Canc Netw. (2021) 19:439–57. doi: 10.6004/jnccn.2021.0017 33845462

[3] Passaro A Al Bakir M Hamilton EG Diehn M Andre F Roy-Chowdhuri S. Cancer biomarkers: Emerging trends and clinical implications for personalized treatment. Cell. (2024) 187:1617–35. doi: 10.1016/j.cell.2024.02.041 PMC7616034 38552610

[4] Perri G Prakash LR Katz MHG. Response to preoperative therapy in localized pancreatic cancer. Front Oncol. (2020) 10:516. doi: 10.3389/fonc.2020.00516 32351893 PMC7174698

[5] Routy B Le Chatelier E Derosa L Duong CPM Alou MT Daillere R. Gut microbiome influences efficacy of PD-1-based immunotherapy against epithelial tumors. Science. (2018) 359:91–7. doi: 10.1126/science.aan3706 29097494

[6] Pernigoni N Zagato E Calcinotto A Troiani M Mestre RP Cali B. Commensal bacteria promote endocrine resistance in prostate cancer through androgen biosynthesis. Science. (2021) 374:216–24. doi: 10.1126/science.abf8403 34618582

[7] Tintelnot J Xu Y Lesker TR Schonlein M Konczalla L Giannou AD. Microbiota-derived 3-IAA influences chemotherapy efficacy in pancreatic cancer. Nature. (2023) 615:168–74. doi: 10.1038/s41586-023-05728-y PMC9977685 36813961

[8] Liu L Shah K. The potential of the gut microbiome to reshape the cancer therapy paradigm: A review. JAMA Oncol. (2022) 8:1059–67. doi: 10.1001/jamaoncol.2022.0494 35482355

[9] Farnes I Kleive D Verbeke C Aabakken L Issa Epe A Småstuen MC. Resection rates and intention-to-treat outcomes in borderline and locally advanced pancreatic cancer - Real-world data from a population-based, prospective cohort study (NORPACT-2). BJS Open. (2023) 7(6). doi: 10.1093/bjsopen/zrad137 PMC10755199 38155512

[10] Braadland PR Bergquist A Kummen M Bossen L Engesaeter LK Reims HM. Clinical and biochemical impact of vitamin B6 deficiency in primary sclerosing cholangitis before and after liver transplantation. J Hepatol. (2023) 79:955–66. doi: 10.1016/j.jhep.2023.05.038 37328069

[11] Midttun Ø. Hustad S Ueland PM. Quantitative profiling of biomarkers related to B-vitamin status, tryptophan metabolism and inflammation in human plasma by liquid chromatography/tandem mass spectrometry. Rapid Commun Mass Spectrometry. (2009) 23:1371–9. doi: 10.1002/rcm.v23:9 19337982

[12] Bjerregaard JK Mortensen MB Schonnemann KR Pfeiffer P. Characteristics, therapy and outcome in an unselected and prospectively registered cohort of pancreatic cancer patients. Eur J Cancer. (2013) 49:98–105. doi: 10.1016/j.ejca.2012.07.017 22909997

[13] Martinez-Jimenez F Movasati A Brunner SR Nguyen L Priestley P Cuppen E. Pan-cancer whole-genome comparison of primary and metastatic solid tumours. Nature. (2023) 618:333–41. doi: 10.1038/s41586-023-06054-z PMC10247378 37165194

[14] Riley RD Snell KI Ensor J Burke DL Harrell FE Jr. Moons KG. Minimum sample size for developing a multivariable prediction model: PART II - binary and time-to-event outcomes. Stat Med. (2019) 38:1276–96. doi: 10.1002/sim.v38.7 PMC6519266 30357870

[15] Roager HM Licht TR. Microbial tryptophan catabolites in health and disease. Nat Commun. (2018) 9:3294. doi: 10.1038/s41467-018-05470-4 30120222 PMC6098093

[16] Song W Sun LY Zhu ZJ Wei L Qu W Zeng ZG. Association of gut microbiota and metabolites with disease progression in children with biliary atresia. Front Immunol. (2021) 12:698900. doi: 10.3389/fimmu.2021.698900 34630385 PMC8495239

