# Supplementary figures and images for: Indole 3-acetate and response to therapy in borderline resectable or locally advanced pancreatic cancer
Source: Front Oncol. 2024 Dec 20;14:1488749. doi: 10.3389/fonc.2024.1488749 (PMC11695226; doi:10.3389/fonc.2024.1488749)

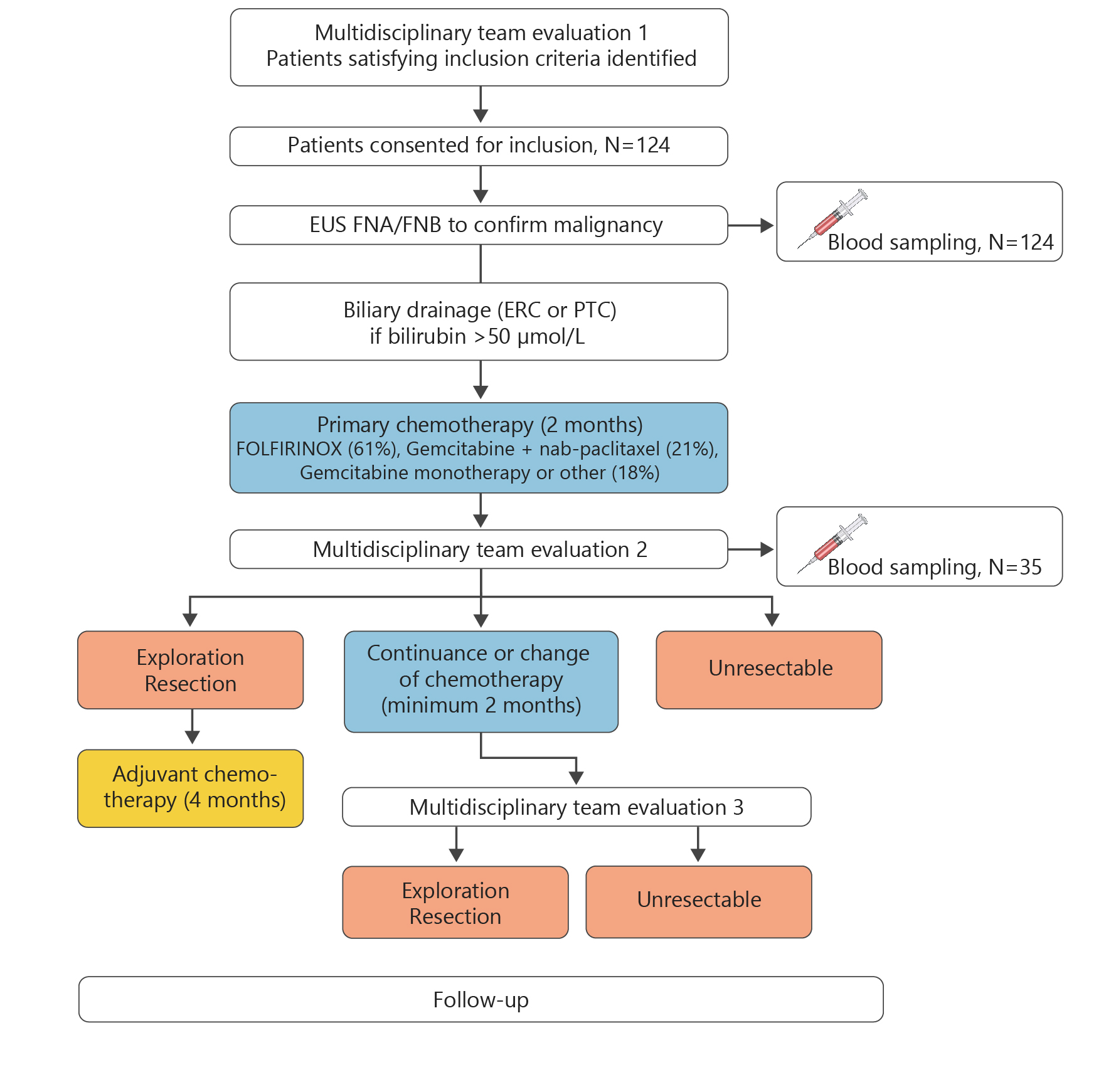

Supplement: Supplementary Figure 1 — Flow chart showing standard-of-care for this patient group and the timing of blood samples in the present study. [file Image1.jpeg]

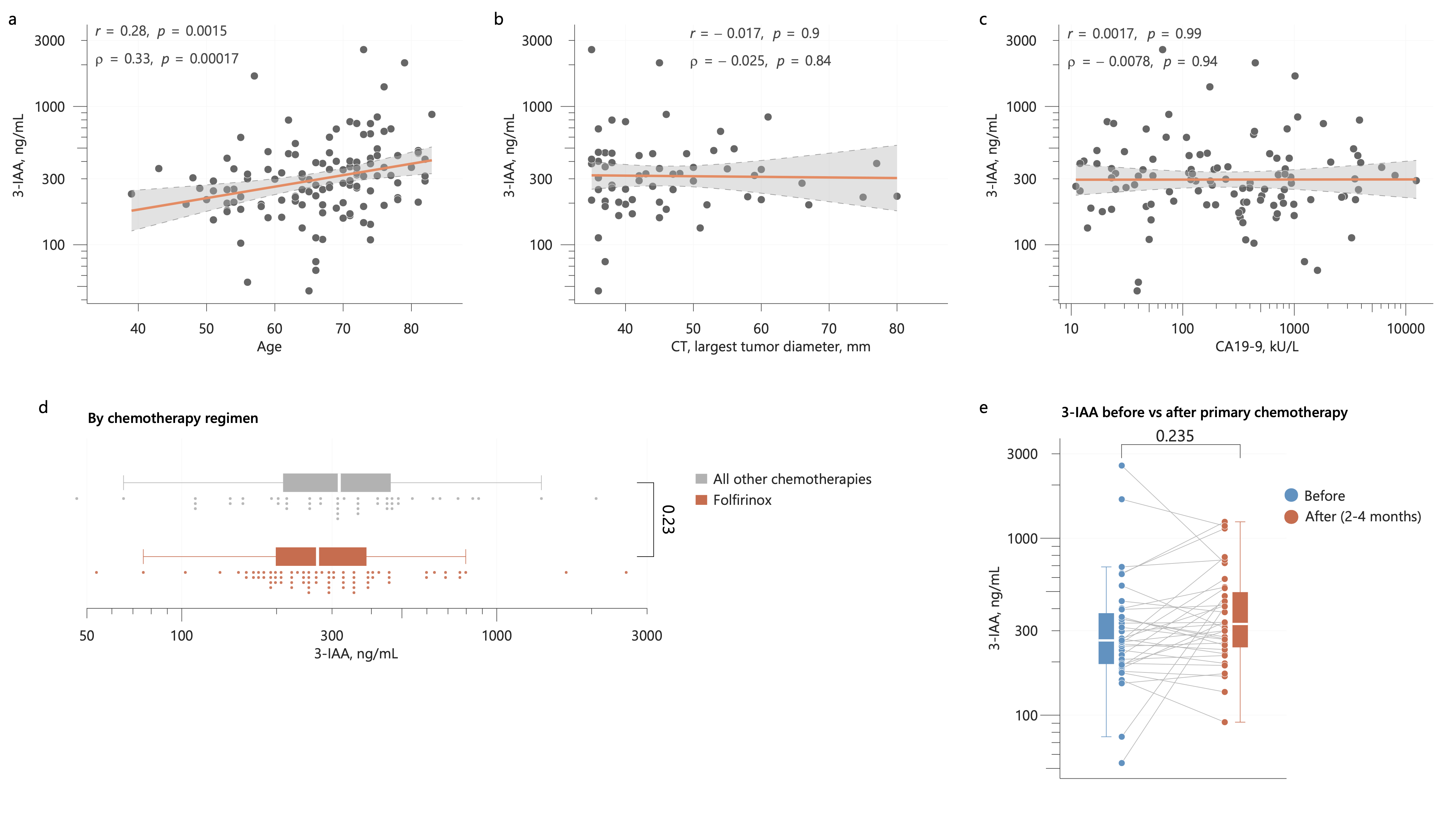

Supplement: Supplementary Figure 2 — (A–C) Plots of baseline 3-IAA concentrations versus age, largest tumor diameter (by CT-scan) and CA19-9, all measured at baseline (before primary chemotherapy). The bivariate associations were tested using Pearson’s (r) and Spearman’s (ρ) correlation. The red lines show the linear regression fits with shaded 95% confidence intervals. (D) Distribution of pre-treatment 3-IAA by chemotherapy regimen (FOLFIRINOX versus all other). Statistical significance was tested using a Mann-Whitney U test. (E). Distributions of 3-IAA before and after primary chemotherapy, with lines indicating changes from before to after. Statistical significance was tested using a paired Wilcoxon signed-rank test. 3-IAA, 3-indoleacetic acid; FFX, FOLFIRINOX. [file Image2.jpeg]

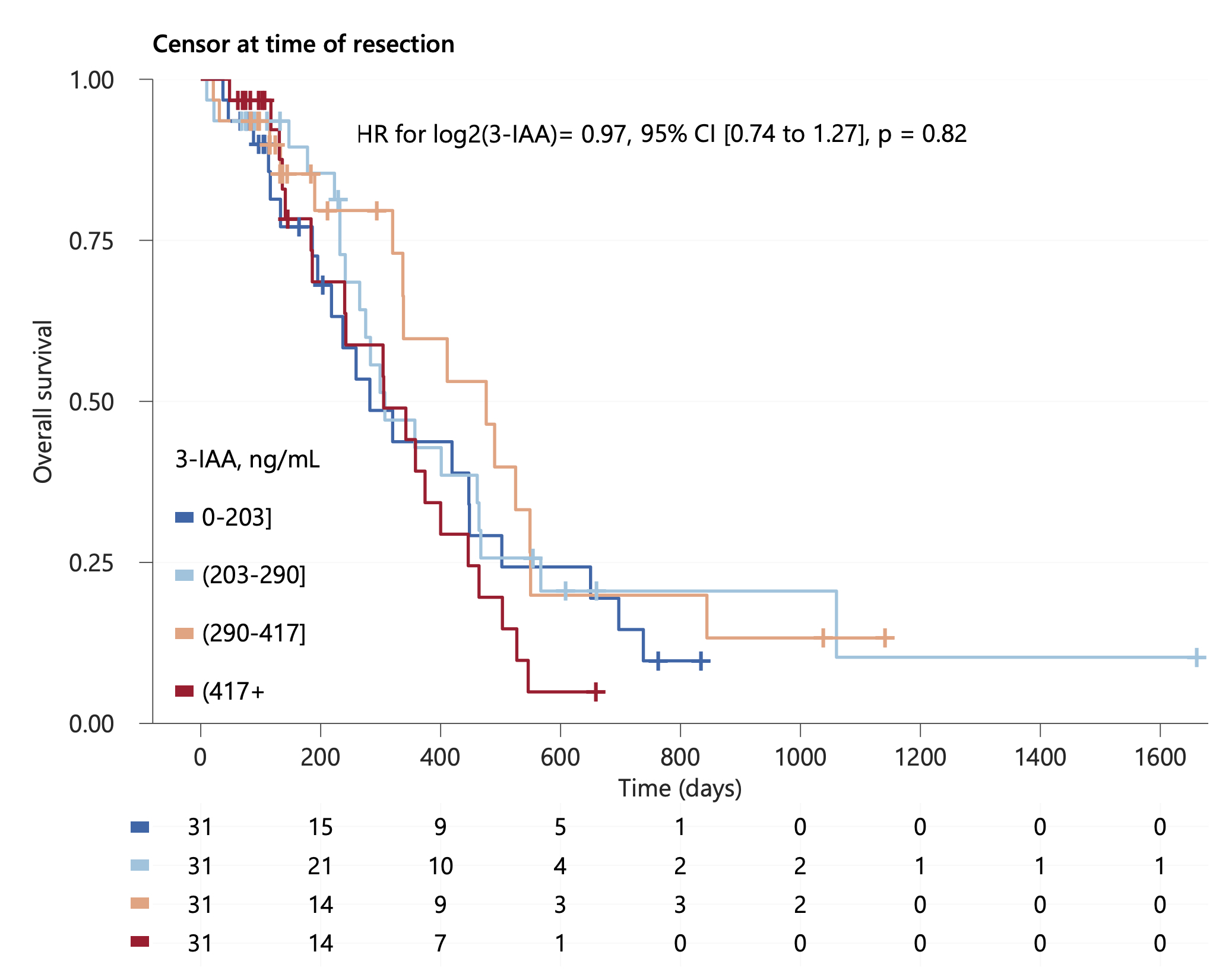

Supplement: Supplementary Figure 3 — Kaplan-Meier survival curves for overall survival, censoring participants who underwent surgical resection at their times of surgery. Patients were categorized by their baseline 3-IAA concentration (quartiles). The number of patients at risk at the indicated time points is shown below the plot. The results from a univariable Cox model for log2(3-IAA) is shown as an inset. 3-IAA, indole 3-acetate; CI, confidence interval; HR, hazard ratio. [file Image3.jpeg]
